# A Case of Atrial Fibrillation from Cyclosporine Toxicity

**Published:** 2004-01-01

**Authors:** Pramod Sanghi, Masood Ahmad

**Affiliations:** Department of Cardiology, University of Texas Medical Branch

**Keywords:** atrial fibrillation, cyclosporine, seizure

## Abstract

We describe the unusual occurrence of atrial fibrillation immediately after a seizure in a young patient with cyclosporine toxicity.  The new onset atrial fibrillation was triggered by high levels of cyclosporine and possibly facilitated by the electrolyte imbalances post seizure and the presence of underlying mild left atrial enlargement.

A twenty-four year old obese (100 kg) man with a past medical history of pure red cell aplasia diagnosed 8 months ago was admitted for elective chemotherapy after having failed outpatient therapy with high dose prednisone and azathioprine. Upon admission, the patient was placed on IV cyclosporine (12 mg/kg/day divided BID) and oral prednisone (1 mg/kg/day).  On the 3rd hospital day, IV anti-thymocyte globulin (4000 mg/day) infused with IV methylprednisolone (125 mg/day) was initiated.  On the 4th hospital day a serum cyclosporine level returned elevated at 995 ng/ml.  At this point the cyclosporine dose was decreased to 8 mg/kg/day. He was also transfused 2 units of leukocyte reduced packed red blood cells for his chronically depressed blood counts (Hemoglobin 6.2 gm/dl). The patient had an uncomplicated hospital course until the 8th hospital day when he was found in his room having a tonic-clonic seizure that lasted approximately 1-2 minutes.  Shortly afterwards, he was found to be in atrial fibrillation (Figure 1) with a ventricular rate of 160 bpm and hypotensive with a blood pressure of 90/50 mmHg.  After a brief postictal period, he denied any complaints but was aware of his tachycardia.  The patient was initially given a loading dose of dilantin, and his heart rate was controlled with boluses of IV diltiazem. Laboratory values taken immediately after his seizure showed a K^+^ of 3.0 mmol/l, Ca^2+^ of 6.7 mg/dl, Mg^2+^ of 1.0 mg/dl, and CO_2_ of 9 mmol/l, but on repeat labs taken 2 hours later, the electrolyte imbalances had all normalized.  The patient’s heart rhythm spontaneously converted to normal sinus (Figure2) about 5 hours after he was initially found to be in atrial fibrillation. His cyclosporine level that had been drawn just a few hours prior to his seizure returned as 1360 ng/ml, which is over 4 times the therapeutic dose for this treatment. The cyclosporine was held and the following day returned as 264 ng/nl.  Echocardiogram performed the following day revealed normal systolic function (LVEF 55 - 60%) with a mildly enlarged left atrium (4.2 cm) and mild to moderate tricuspid regurgitation.  His estimated pulmonary artery pressure was 40 mmHg.

## Discussion

Although this is not the first time atrial fibrillation has been reported in the literature from an overdose of cyclosporine [1], our case is unusual for the following reasons. First of all, our patient received an appropriate dose of cyclosporine but was still found to have toxic levels of the drug. While he was given the correct dosages for his weight (12 kg/mg/day), the patient was obese and some studies suggest that the dosages of cyclosporine in these patients not be based on their actual body weight [2]. Other potential causes of high blood cyclosporine levels in this patient are increased gastrointestinal absorption secondary to high dietary fat intake while in the hospital or an idiosyncratic drug reaction [3].

What is also unique about this case is that the patient suffered a seizure prior to the onset of atrial fibrillation. Seizure is a well described complication of cyclosporine toxicity [4]. The electrolyte imbalances assumed to be secondary to the seizure may have predisposed the patient to atrial fibrillation in the face of cyclosporine toxicity. Although atrial fibrillation is a known complication of cyclosporine toxicity independent of seizure activity or electrolyte imbalances, the atrial fibrillation seen in this case occurred at a relatively low level of cyclosporine toxicity as compared to other case reports in the literature [1]. In addition, the patient may have been at further risk of developing atrial fibrillation due to the mild left atrial enlargement seen on his echocardiogram.

 Although the mechanism by which cyclosporine causes atrial fibrillation is unknown, cyclosporine is known to have some deleterious cardiac effects including coronary vasospasm [5] and ventricular hypertrophy [6]. Further research is needed to determine the mechanisms by which these potentially harmful cardiac effects occur.

## Figures and Tables

**Figure 1:**
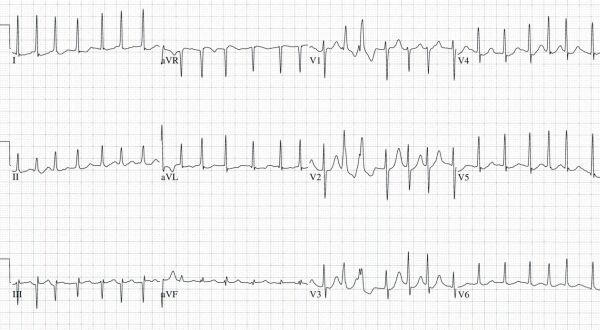
Electrocardiogram showing atrial fibrillation in a 24 year old man taken immediately after a witnessed seizure.  His cyclosporine level was toxic at 1360 ng/ml.

**Figure 2:**
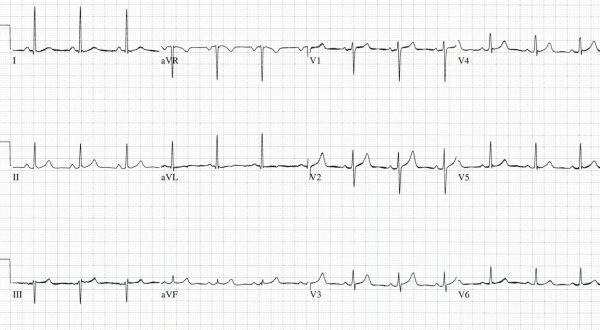
Electrocardiogram showing normal sinus rhythm taken the following morning after the initial onset of atrial fibrillation.  The cyclosporine level drawn at this time was 264 ng/ml.
